# Validation of a Fast and Simple HPLC-UV Method for the Quantification of Adenosine Phosphates in Human Bronchial Epithelial Cells

**DOI:** 10.3390/molecules26206324

**Published:** 2021-10-19

**Authors:** Ana Teresa Juarez-Facio, Violaine Martin de Lagarde, Christelle Monteil, Jean-Marie Vaugeois, Cécile Corbiere, Tiphaine Rogez-Florent

**Affiliations:** Normandie Univ, UNIROUEN, UNICAEN, ABTE, 76000 Rouen, France; ana-teresa.juarez-facio1@etu.univ-rouen.fr (A.T.J.-F.); violaine.martin-de-lagarde@univ-rouen.fr (V.M.d.L.); christelle.monteil@univ-rouen.fr (C.M.); jean-marie.vaugeois@univ-rouen.fr (J.-M.V.); cecile.corbiere@univ-rouen.fr (C.C.)

**Keywords:** HPLC-UV, BEAS-2B cells, NHBE cells, adenosine phosphates, cellular extraction

## Abstract

A new HPLC method for the simultaneous quantitative analysis of adenosine triphosphate (ATP), adenosine diphosphate (ADP), and adenosine monophosphate (AMP) was developed and validated. ATP, ADP, and AMP were extracted from human bronchial epithelial cells with a rapid extraction procedure and separated with a C18 column (3 × 150 mm, 2.7 µm) using isocratic elution with a mobile phase consisting of 50 mM of potassium hydrogen phosphate (pH 6.80). The absorbance was monitored at 254 nm. The calibration curves were linear in 0.2 to 10 µM, selective, precise, and accurate. This method allowed us to quantify the nucleotides from two cell models: differentiated NHBE primary cells grown at the air–liquid interface (ALI) and BEAS-2B cell line. Our study highlighted the development of a sensitive, simple, and green analytical method that is faster and less expensive than other existing methods to measure ATP, ADP, and AMP and can be carried out on 2D and 3D cell models.

## 1. Introduction

Adenosine 5′-triphosphate (ATP) is a key regulator of several cellular functions such as synthesis of nucleic acids and many biochemical processes including cell energy metabolism [1] as well as tumor energy metabolism [2]. Its measure is commonly used for cell viability and cytotoxicity in research and drug discovery. Quantifying other nucleotides such as adenosine 5′-diphosphate (ADP) and adenosine 5′-monophosphate (AMP) is important to have an overview of the cellular energy state and allows a better understanding of the mechanisms that lead to cell damage or death [3,4]. However, measurement of nucleotides in cell cultures may be a difficult task as samples are frequently in low concentrations [5,6].

ATP level is frequently determined by assay kits based on enzymatic bioluminescence or fluorescence detection [7]. Kits rarely concern ADP and AMP nucleotides and they could become very expensive through time. Recently, the new technology of Seahorse analyzers appeared [8]. It measures real-time ATP levels in living cells, providing valuable information on cellular bioenergetics such as mitochondrial respiration and glycolysis. However, this technology is expensive and does not allow the quantification of ADP and AMP. To quantify ADP and AMP, instrumental analytical techniques have been developed, including chromatographic methods such as ion exchange, thin-layer and High-Performance Liquid Chromatography (HPLC) often with organic solvents used for mobile phase, which are endowed with high sensitivity and efficiency [9].

In this study, we proposed a sensitive method to quantify ATP, ADP, and AMP adenosines by HPLC-UV with little sample preparation and water as buffer for mobile phase. Efficient extraction of adenosine nucleotides in two bronchial epithelial cell models was also established. Extraction and measurement methods were validated concerning linearity, sensitivity, reproducibility, and precision.

## 2. Results

### 2.1. Method Optimization

**Figure 1:**
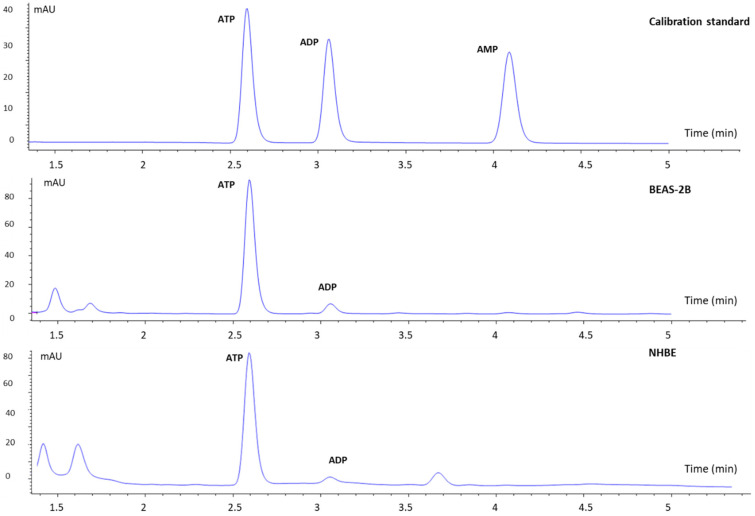
Representative overlaid HPLC chromatograms of calibration standard (X), BEAS-2B cells sample, differentiated NHBE cells sample. Poroshell 120 EC-C18 (3 × 150 mm, 2.7 µm), 20 °C, 50 mM of potassium hydrogen phosphate (pH 6.80), 0.6 mL/min, 254 nm.

The methodology proposed in this work aimed to develop a rapid method for quantification of adenosine phosphates in bronchial epithelial cells. Different columns were tested to develop the chromatographic separation of the three adenosine phosphates, ATP, ADP, and AMP. The first one was a porous graphite carbon column (Hypercarb, 2.1 mm × 100 mm × 5 µm), but ATP and ADP could not be separated on the column. Next, two HILIC columns were tested, a Nucleoshell HILIC (4.6 mm × 150 mm, 2.7 µm) and a Uptisphere Strategy (2,1 mm × 150 mm, 3 µm). Although both columns showed good resolution, peak tailing was also obtained. In addition, the column backpressure rapidly increased when using the Uptisphere column, resulting in a poor column lifespan after a limited number of sample solution injections. The Poroshell 120 EC-C18 column (3 mm × 150 mm, 2.7 µm) was then used. Several chromatographic parameters, such as phosphate buffer concentration, pH, temperature, and flow rate were evaluated to better separate the three adenosine phosphates with a minimum of run time. The three adenosine phosphates were successfully separated. Hence, the best results were obtained in isocratic mode with 50 mM phosphate buffer at pH 6.8. Figure 1 shows the resulting chromatograms.

### 2.2. Method Validation

The developed HPLC-UV method was passed through the following validation parameters: selectivity, linearity, the limit of detection (LOD) and quantification (LOQ), accuracy, and precision.

#### 2.2.1. Selectivity

In biological complex matrices such as bronchial epithelial cells, it is particularly relevant to study the method’s selectivity. In all analyses, no interfering peak at the same retention time of the analytes was detected, proving the method’s selectivity. As the biological samples may easily contaminate the C18 column, reducing the column’s life span, in the present study, C18 column was washed 30 min with water 100% and 30 min with a mixture of water/ACN (70/30) after the injections of the day. Thus, the column’s life span was preserved (changing the guard column every 500 injections).

#### 2.2.2. Linearity, the Limits of Detection and Quantification

Calibration curves were calculated using peak areas at six standard concentrations. Linear regression of ATP, ADP, and AMP was obtained for all analytes from the studied concentration range. The regression coefficient for all calibration curves was greater than 0.999 (Table 1). The standard solutions were diluted gradually and analyzed to ascertain the limit of detection (LOD), and the limit of quantification (LOQ), defined as signal-to-noise ratios (S/N) of 3:1 and 10:1, respectively (Table 1).

#### 2.2.3. Precision and Accuracy

The results of within-day and between-day accuracy and precision are summarized in Table 2. The coefficients of variation (CV%) for the within-day precision ranged from 0.2% to 2.6%, while the between-day precision values ranged from 0.5% to 8.7%. Accuracy was expressed as the bias %. Intra-day accuracy ranged from 0.1% to 11.3%, and inter-day accuracy ranged from 0.1% to 3.7% for an analyte.

#### 2.2.4. Stability

The stability of the cell samples spiked of ATP, ADP, and AMP were determined after injecting the three extracts at six different times. After 24 h, coefficients of variation of ATP, ADP, and AMP were less than 10%.

Concerning the stability of ATP, ADP, and AMP standard solution in the mobile phase, after three freezing–thawing cycles, no degradation was observed.

#### 2.2.5. Standard Substance Spiked in Recovery

A known concentration of ATP, ADP, and AMP (10 µM) was spiked to cell extract with known concentration and was analyzed. The method’s recovery after addition of known amounts of analytes to cell samples ranged between 97.8% and 110.5%.

### 2.3. Extraction Optimization

The validated HPLC method was used to evaluate the method’s relevance for ATP, ADP, and AMP quantification in a human bronchial epithelial cell model (BEAS-2B). The extraction protocol was based on Barraud et al. [10]. After addition of PCA, the sample was centrifuged to remove proteins and cell debris, and finally, after neutralization with K_2_CO_3_, perchlorates were removed by centrifugation. In order to develop an optimal and standardized extraction protocol, different conditions of metabolite extraction were performed. First, we performed extraction by scratching the cells directly in PCA (PCA extract) or by resuspending the cell pellet in PCA. Then, we analyzed both conditions directly after extraction and after freezing the samples.

**Figure 2:**
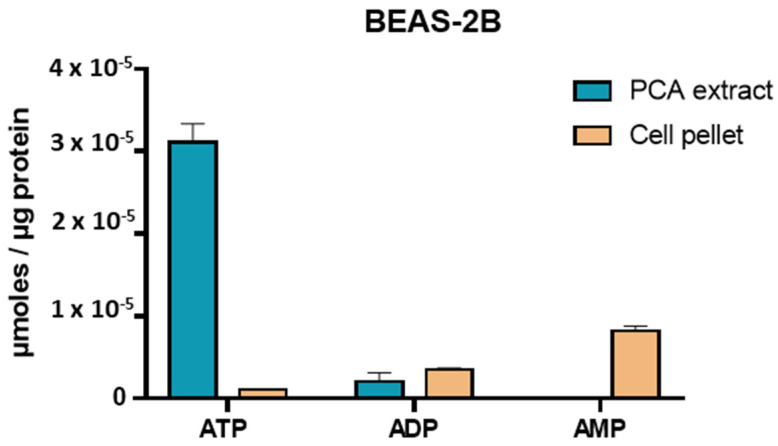
ATP, ADP, and AMP levels (µmol/µg protein) in BEAS-2B cells (*n* = 4 independent experiments).

Figure 2 shows the effects of sample extraction on ATP, ADP, and AMP concentrations in BEAS-2B cells. ATP concentration from PCA extract (3.1 × 10^−5^ µmol/µg protein) was higher than extraction from the cell pellet (1 × 10^−6^ µmol/µg protein). Indeed, extraction from the cell pellet induced degradation of ATP into ADP and AMP, whereas this effect was not observed in direct PCA extraction. Our results indicated that the extraction method had a significant impact on metabolite dosage since extraction from the cell pellet resulted in a loss of information and degradation of metabolites. Therefore, direct extraction in PCA was chosen as the optimal extraction protocol to dose ATP, ADP, and AMP. Concerning metabolites quantification on fresh extract and frozen extract, we observed no difference in ATP concentration (frozen extract, ATP: 2.7 × 10^−5^ µmol/µg protein; fresh extract, ATP: 2.6 × 10^−5^ µmol/µg protein; *n* = 3), allowing us to work with frozen samples. According to these data, this protocol can be applied to experimental protocols carried out over several days, with many samples, or in a place remote from the analysis laboratory.

### 2.4. Application to a 3D Bronchial Epithelial Cell Model (NHBE)

The protocol’s extraction and the method optimized to quantify ATP, ADP, and AMP were applied on a second human bronchial epithelial cell model: NHBE cells which were differentiated into a mucociliary phenotype at the air–liquid interface. The contents of ATP, ADP, and AMP in the samples were calculated according to the external-standard calibration curves. In addition, ATP/ADP ratio was calculated. The results are summarized in Table 3 and showed that this method could be applied to a complex/3D cellular model.

## 3. Discussion

HPLC has been described as a highly sensitive separative method that allows the separation and simultaneous quantification of a wide range of nucleotides [11,12,13,14,15]. Several HPLC-UV studies have been performed to quantify ATP, ADP, and AMP in biological matrices (bacteria, cells, biomass, tissue, erythrocytes…) [11,12,13]. To our knowledge, few studies employing HPLC-UV are reported to determine ATP, ADP, and AMP concentration in complex cell models [9]. In the present study, we optimized a simple and rapid reversed-phase HPLC method to determine ATP, ADP, and AMP, in bronchial epithelial cells. The run time was less than 4.5 min, whereas the reported methods (HPLC-UV, reverse phase: C18, ATP/ADP/AMP, biological matrix) have a run time between 8 and 40 min [11,13]. In addition, our analysis was an isocratic method, unlike the reverse-phase methods described [11,12]. This avoided waste of time between injections and the stabilization that usually takes 10 min. The LOD and LOQ were close to or even higher than those described in the literature [11]. The proposed method was environmentally friendly. Indeed, the absence of solvents in the mobile phase and in the extraction protocol enabled a green analytical method for the quantification of ATP, ADP, and AMP in a biological matrix. In addition, a simple and fast extraction protocol was also established. Furthermore, the quantification of adenosine phosphates can be determined inexpensively with routine classic equipment available in many laboratories. It should be noted that the rinsing procedure after analysis extended the column’s life and the column’s guard. Under these analytical conditions, more than 500 injections were performed with the same guard column. It should be noted that the cost per sample was lower than commercial assay kits. Moreover, it was interesting to analyze the three adenosines nucleotides simultaneously for the accurate measure of cellular energy charge (EC = [ATP] + 0.5 [ADP]/[ATP] + [ADP] + [AMP]) and study the changes in energy metabolism after exposure to xenobiotics.

## 4. Materials and Methods

### 4.1. Chemicals and Reagents

Adenosine 5′-triphosphate disodium salt hydrate (ATP, 99%), adenosine 5′-diphosphate sodium salt (ADP, 98%), 5′-monophosphate sodium salt (AMP, 99%), potassium dihydrogen phosphate (99%), dipotassium hydrogen phosphate (99%), potassium carbonate (99%), and perchloric acid (PCA) (70%) were purchased from Sigma–Aldrich Company (St. Louis, MO, USA). Ultra-pure water was supplied by a Micropure UV purification system from Cloup (Champigny, France).

### 4.2. Instrumentation and Chromatographic Conditions

Chromatographic analyses were performed using an Agilent High-Pressure Liquid Chromatography system 1260 Infinity II equipped with a quaternary pump, an autosampler, and a photodiode array detector (Agilent Technologies, Santa Clara, CA, USA). The system control and data processing were performed using an OpenLab CDS LC ChemStation from Agilent. Separation was carried out on a reverse-phase Poroshell 120 EC-C18 (3 × 150 mm, 2.7 µm) with guard EC-C18 (3 mm) maintained at 20 °C. The injection volume was 1 µL. The compounds in standard and sample solutions were separated using a mobile phase consisting of 50 mM of potassium hydrogen phosphate (100%, pH 6.80) at 0.6 mL/min. The working solutions were prepared daily by appropriate dilutions with the mobile phase. The absorbance was monitored at 254 nm.

### 4.3. Standard Solution Preparation

Stock solutions of 10 mM ATP, ADP, and AMP were prepared by dissolving the appropriate amounts of these compounds in ultra-pure water, and stored in aliquots at −20 °C.

### 4.4. Cell Culture and Sample Extraction

#### 4.4.1. Cell Culture

The human bronchial epithelial cell line BEAS-2B (ATCC) and primary NHBE cells (ATCC) were used. Both cell models were cultured in submerged conditions for 4 days in LHC-9 medium (GIBCO) for BEAS-2B, and airway epithelial cell basal medium (ATCC) for NHBE, both supplemented with 10 U/mL penicillin and 10 µg/mL streptomycin. Between 80% and 90% confluence, the cells were harvested and then seeded on collagen (30 µg/mL)-coated transwell^TM^ inserts at a density of 90,000–100,000 cells/insert. BEAS-2B culture was maintained at the liquid–iquid interface for 10 days while NHBE was cultured at the air–liquid interface (ALI) with pneumacult-ALI basal medium (StemCell) for 14 days. The ALI culture of NHBE cells forms a pseudostratified epithelium that exhibits morphological and functional characteristics similar to those of the human airway in vivo [16]. Then, cells extraction was carried out. For both cell models, the medium was changed every two days, and the cultures were maintained in 95% humidified air with 5% CO_2_ at 37 °C. 

#### 4.4.2. Sample Extraction

Globally, 500 µL of PCA (1 N) was added to samples (cell layer or cell pellet) then briefly vortexed. Then, samples were centrifuged at 13,500× *g* for 5 min at 4 °C to precipitate proteins. Next, 250 µL of K_2_CO_3_ (2 M) was added to the supernatant to neutralize it. Samples were then centrifuged again, and supernatants were collected and stored at −80 °C until metabolite measurement. Protein pellets were collected in NaOH (1 M), and metabolites dosage was normalized to protein content measured by Lowry assay.

### 4.5. Method Validation

The method was validated in terms of selectivity, linearity, the limit of quantification, accuracy, and precision, according to the ICH Q2(R1) guidelines [17]. The standard solutions were diluted gradually and analyzed to obtain the peak signal-to-noise of at least 3 and 10, at which the limit of detection (LOD) and the limit of quantification (LOQ) were, respectively, produced. The validation protocol used was based on a three-day validation method, each day, a calibration curve consisting of six calibration standards (CS), from 0.2 to 10 µM for ATP, ADP, and AMP. Four quality control samples (QC) (LOQ, 3 × LOQ, and 50% and 75% of the last points of the calibration curve) in triplicate were analyzed. The linearity of the calibration curve was determined by the R^2^ coefficient, and intra-day and inter-day precision were achieved using the coefficient of variation (CV%), and the relative bias from the error of the theoretical value calculation. To test the stability of the samples, the cell samples spiked 10 µM of ATP, ADP, and AMP in triplicate and were injected after 0, 6, 12, and 24 h by storing the sample in brown glass vials in a thermostated injector at 15 °C. The coefficient of variation was also determined.

## 5. Conclusions

This study described a simple, sensible, durable, environmentally friendly, and fast method for low-level quantification of ATP, ADP, and AMP using HPLC-UV in bronchial cells. The three adenosine phosphates were successfully separated with a reversed-phase C18 column with a short run time at 4.5 min. Regarding extraction optimization, several parameters were studied. Our results demonstrated that direct PCA extraction is better than extraction from the cell pellet because of its ability to measure the three adenosine phosphates with no degradation. In addition, the established protocol can be applied on multiple cell models, cell lines, or primary cells cultivated in 2D or 3D. This protocol can be used to validate rapidly experimental conditions (diluent effect, air-flow effect, for example) and to study the changes in energy metabolism after exposure to xenobiotics such as chemical or particulate matter as well as drugs.

## Figures and Tables

**Table 1:** Linear regression, LOD, LOQ, and recovery for ATP, ADP, and AMP.

Analytes	Calibration Curve	Correlation Coefficient R^2^	Linear Ranger (µM)	LOD (µM)	LOQ (µM)	Recovery (%)	CV%
ATP	y = 35.21x − 0.7885	0.9999	0.2–10	0.054	0.18	110.4	2.9
ADP	y = 28.9x − 0.1143	0.9999	0.2–10	0.060	0.20	97.8	8.1
AMP	y = 30.5x − 0.5111	0.9999	0.2–10	0.051	0.17	110.5	5.1

**Table 2:** Intra-day and inter-day accuracy (Bias%) and precision (CV%) of ATP, ADP, and AMP.

Analytes	Concentration (µM)	Intra-Day (*n* = 3)			Inter-Day (*n* = 9)		
		Mean Measured Concentration (µM) ± S.D.	Bias%	CV%	Mean Measured Concentration (µM) ± S.D.	Bias%	CV%
ATP	0.2	0.22 ± 0.001	11.3	0.7	0.20 ± 0.02	0.1	8.7
	0.6	0.63 ± 0.003	4.3	0.5	0.61 ± 0.01	2.2	2.1
	5	5.00 ± 0.016	0.8	0.4	5.03 ± 0.03	0.7	0.5
	7.5	7.42 ± 0.194	1.1	2.6	7.50 ± 0.13	0.1	1.7
ADP	0.2	0.20 ± 0.006	1.9	3.1	0.19 ± 0.01	3.7	4.8
	0.6	0.60 ± 0.003	0.2	0.5	0.60 ± 0.01	0.3	1.1
	5	4.95 ± 0.015	1.1	0.3	4.97 ± 0.02	0.5	0.5
	7.5	7.29 ± 0.193	2.7	2.6	7.44 ± 0.15	0.9	1.7
AMP	0.2	0.22 ± 0.001	8.1	0.2	0.20 ± 0.01	0.2	6.5
	0.6	0.57 ± 0.020	2.5	0.6	0.61 ± 0.01	2.4	0.5
	5	5.00 ± 0.020	0.1	0.4	5.06 ± 0.06	1.1	1.2
	7.5	7.37 ± 0.195	1.8	2.7	7.52 ± 0.16	0.3	2.2

**Table 3:** Determination of ATP, ADP, AMP in NHBE cells (µmol/µg protein).

Sample No.	ATP	ADP	AMP	ATP/ADP
1	3.90 × 10^−5^	1.43 × 10^−6^	7.91 × 10^−7^	27.3
2	3.43 × 10^−5^	1.26 × 10^−6^	1.47 × 10^−7^	27.2
3	3.40 × 10^−5^	1.22 × 10^−6^	1.76 × 10^−6^	27.9
4	3.52 × 10^−5^	1.26 × 10^−6^	7.04 × 10^−7^	27.9
Mean	3.56 × 10^−5^	1.29 × 10^−6^	8.51 × 10^−7^	27.6
SD	2.31 × 10^−6^	9.36 × 10^−8^	6.70 × 10^−7^	0.4
